# TLR4 signalling in pulmonary stromal cells is critical for inflammation and immunity in the airways

**DOI:** 10.1186/1465-9921-12-125

**Published:** 2011-09-24

**Authors:** Frederic Perros, Bart N Lambrecht, Hamida Hammad

**Affiliations:** 1 Laboratory of Immunoregulation and Department of Respiratory Medicine, University Hospital of Ghent, 185 De Pintelaan, Ghent, B-9000, Belgium; 2 Université Paris-Sud, Faculté de médecine, 63 Rue Gabriel Péri, Le Kremlin-Bicêtre, F-94276, France

**Keywords:** Airway diseases, dendritic cells, epithelial cell, pulmonary stromal cells, TLR4

## Abstract

Inflammation of the airways, which is often associated with life-threatening infection by Gram-negative bacteria or presence of endotoxin in the bioaerosol, is still a major cause of severe airway diseases. Moreover, inhaled endotoxin may play an important role in the development and progression of airway inflammation in asthma. Pathologic changes induced by endotoxin inhalation include bronchospasm, airflow obstruction, recruitment of inflammatory cells, injury of the alveolar epithelium, and disruption of pulmonary capillary integrity leading to protein rich fluid leak in the alveolar space. Mammalian Toll-like receptors (TLRs) are important signalling receptors in innate host defense. Among these receptors, TLR4 plays a critical role in the response to endotoxin.

Lungs are a complex compartmentalized organ with separate barriers, namely the alveolar-capillary barrier, the microvascular endothelium, and the alveolar epithelium. An emerging theme in the field of lung immunology is that structural cells (SCs) of the airways such as epithelial cells (ECs), endothelial cells, fibroblasts and other stromal cells produce activating cytokines that determine the quantity and quality of the lung immune response. This review focuses on the role of TLR4 in the innate and adaptive immune functions of the pulmonary SCs.

## TLRs and TLR4 signalling at a glance

Cytokines that stimulate the innate immune response are not constitutively expressed but must be called into play by specific signals that alert the host to invading micro-organisms. Mammalian Toll-like receptors (TLRs) are similar in structure and function to the Drosophila Toll protein [1]. The cytoplasmic domain of this transmembrane protein is similar to that of the mammalian IL-1 receptor, suggesting that both Toll and mammalian TLRs share similar signal-transduction pathways via a MyD88-dependent pathway that ultimately involves the NF-κB family of transcriptional factors. NF-κB serves as a master switch, transactivating various cytokines that are involved in the innate and transition to adaptive immunity [2]. Medzhitov and colleagues were the first to characterize a human TLR, TLR4 [3]. The constitutively active mutant of TLR4, when transfected into human cell lines, activates NF-κB and stimulates the expression of the proinflammatory cytokines IL-1, -6, and -8. In addition, TLR4 signal transduction and NF-κB transactivation induces expression of IL-12p40, as well as CD80 and CD86, costimulatory molecules that link innate and adaptive immune responses by activating antigen-specific responses by naive T cells. The response to lipopolysaccharide (LPS), a cell wall component of Gram-negative bacteria, is initiated upon its interaction with TLR4 in conjunction with the accessory molecules MD-2 and soluble or membrane-bound CD14 [4]. The response is then transduced via the interleukin (IL)-1 receptor signalling complex, which includes two essential adaptor proteins, myeloid differentiation (MyD)88 and tumor necrosis factor receptor-associated factor (TRAF)6 as well as the serine-threonine kinase known as IL-1R-associated kinase (IRAK). Other components involved in this signalling pathway include mitogen-activated protein kinases (MAPKs) such as extracellular signal-regulated kinase 1/2 (ERK1/2), c-Jun N-terminal kinase (Jnk), and p38 kinase (p38) [5,6]. This signal transduction pathway further coordinates the induction of multiple genes encoding inflammatory mediators and co-stimulatory molecules [7]. A detailed description of the TLR signalling has been reviewed recently [8].

**Figure 1:**
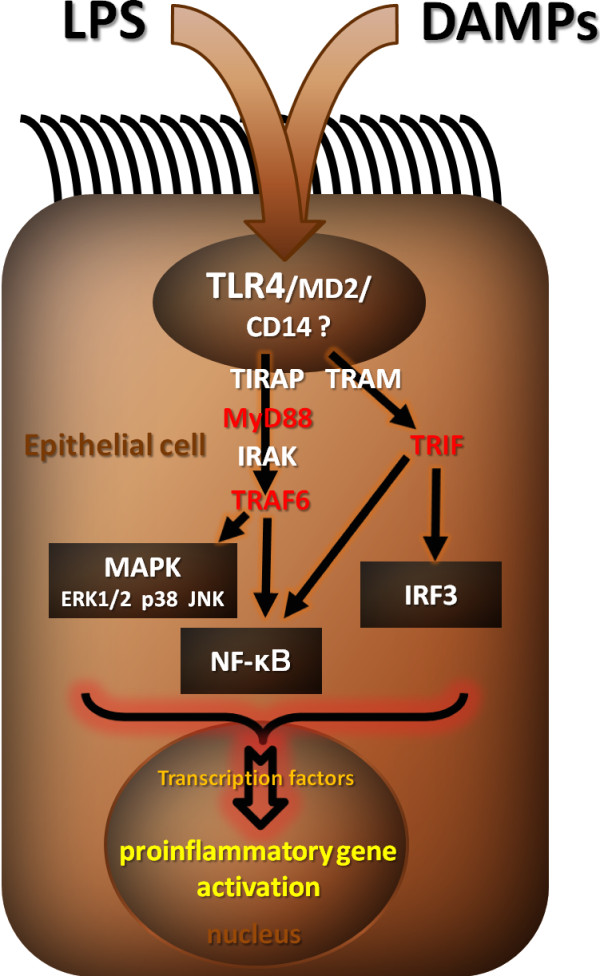
**TLR4 signalling**. Of all the radioresistant stromal cells (SCs), epithelial cells (ECs) that line the airways are the most likely to mediate the effects of LPS, given their exposed position, their known and confirmed expression of TLR4 and their activation of TLR4 dependent signalling cascades upon exposure to TLR4 ligands (LPS, DAMPs, HDM). The intracellular compartmentalization of TLR4 may prevent "inopportune" activation of pulmonary ECs. Whereas TRL4 signalling via MyD88 is essential for the LPS-induced acute pulmonary inflammation response, TRL4 signalling via TRIF is dispensable [9]. MyD88 and TIRAP are involved in early activation of NF-κB and MAPK, whereas TRIF and TRAM are critical for late activation of NF-κB as well as the activation of IRF-3 [9]. There is no consensus about the expression and role of CD14 in LPS-induced lung epithelial activation.

Noulin et al [9] analyzed the role of TLR signalling and the contribution of different cell types in response to aerogenic LPS. They focused on the role of the common TLR and IL-1R adaptor molecule, the MyD88. Absence of MyD88 confers resistance to systemic endotoxin-induced shock [10], although there is evidence that LPS can use MyD88-independent signalling pathways [11]. In particular, other adaptor proteins such as TIR domain-containing adaptor inducing IFN-β (TRIF)3 [12,13] and TRIF-related adaptor molecule (TRAM) [14,15] have been implicated in some responses to LPS resulting in IFN type I-dependent expression of costimulatory molecules. TRAM is thought to act as a link between TRIF and TLR4, like Toll/IL-1R domain-containing adaptor protein (TIRAP) bridging MyD88 to TLR4. MyD88 and TIRAP are involved in early activation of NF-κB and MAPK [16-19], whereas TRIF and TRAM are critical for late activation of NF-κB as well as the activation of IRF-3 [15,20]. A recent work on macrophages/dendritic cells (DC) suggests that no pathway other than MyD88-dependent or TRIF-dependent pathways exists in response to LPS in TLR4-mediated signalling [21], whereas a third pathway independent of TLR4 possibly exists [22]. Noulin et al demonstrated that MyD88 is indeed essential for the LPS-induced acute pulmonary inflammation response, whereas TRIF is dispensable. Accordingly, Guillot et al [23] showed that ECs response to LPS involves at least the signal-transducing molecules MyD88, IRAK, and TRAF6 and activation of the transcription factor NF-κB. Also MAPKs appear to be important mediators of this cell activation process as three of these kinases (p38, Jnk, and ERK1/2) are selectively activated in a time-dependent manner by LPS (Figure 1).

## TLR4 expression in pulmonary stromal cells

Various studies have provided evidence that TLR4 plays a critical role in myeloid cells [24-26], but recent reports suggest that a LPS signalling system also exists in cells of epithelial origin. TLR4 is expressed in intestinal [27,28], renal [29], colonic, and gingival epithelia [30]. In the lung, TLR4 expression has been demonstrated in alveolar and bronchial epithelial and vascular endothelial cells [23,31,32].

Sha et al demonstrated that ECs express mRNA for all TLR and that several known TLR ligands activate epithelial cells to express chemokines, cytokines, and host defense molecules, including acute phase proteins and complement proteins. Moreover the expression of these receptors may be increased by cell activation. Among the induced genes were macrophage inflammatory protein (MIP)-3α and granulocyte macrophage-colony-stimulating factor (GM-CSF), which would be expected to recruit and activate immature DCs that might be important in early triggering of adaptive immune responses. Guillot et al [23] demonstrated by reverse transcription-PCR and/or immunoblot that TLR4 and the accessory molecule MD-2 are constitutively expressed in distinct human alveolar and bronchial ECs. Based on flow cytometry experiments, they showed that is unlikely that LPS might recruit TLR4 to the cell surface upon cell activation. However, it can not be excluded that inflammatory mediators such as cytokines or bioactive lipids might be able to induce TLR4 relocalization. Intracellular compartmentalization of TLR4 allowed nevertheless LPS to strongly induce the secretion of proinflammatory mediators. Epithelial activation by LPS does not alter TLR4 expression at the mRNA or protein level or alter its intracellular localization. In agreement with the absence of TLR4 expression on the cell surface of pulmonary ECs, it was also relevant to notice that addition of a blocking anti-TLR4 antibody in the extracellular medium had no effect on activation by LPS as assessed by the measurement of IL-8 secretion. One can speculate that the intracellular compartmentalization of TLR4 may prevent "inopportune" activation of pulmonary ECs due to a regular exposure to air containing trace amounts of LPS and as a consequence a chronic inflammatory state (Figure 1). In the context of this distinctive cell distribution, TLR4 signalling may therefore be triggered only upon exposure to a high amount of free or bacteria-associated LPS as occurs in occupational or infectious diseases [33,34]. Subsequently the pulmonary epithelium may then participate in the local innate response through the secretion of cytokines and antimicrobial peptides. Interestingly, before the identification of TLR4 as an essential participant in LPS signalling, Wright and colleagues [35] showed that LPS is rapidly delivered from the plasma membrane to an intracellular site and that agents that block vesicular transport alter cell responses to LPS. Moreover Vasselon et al. [36] demonstrated that monomeric LPS crosses the cell membrane and traffics within the cytoplasm independently of membrane CD14, while aggregates of LPS are internalized in association with CD14. However, Guillot et al [23] failed to detect CD14 protein expression in human primary polarized bronchial ECs using confocal microscopy, and no CD14 protein staining could be detected in lung epithelial samples. A similar result was observed using the pulmonary EC line A549 but was not seen with BEAS-2B cells, which express a low level of CD14. Thus, these data do not currently dissipate the debate that exists concerning the expression and role of CD14 in LPS-induced lung epithelial activation. Several authors proposed that these cells are CD14-negative [37,38], while others demonstrated both CD14 mRNA and cell surface protein in human airway ECs [33,39,40]. In fact, these contradictory results may be explained by distinct basal activation or differentiation state of the ECs used throughout these investigations.

Expression of TLR4 in non-BM cells appears to be essential for neutrophil recruitment to the lungs following systemic LPS administration [32]. Andonegui et al. [32], showed that TLR4-deficient neutrophils were sequestered in capillaries of mice expressing TLR4 in non-BM cells within 4 h of intraperitoneal injection of LPS, and the authors speculated that TLR4 expression in the endothelium was required for this recruitment.

## Endotoxin sensing by pulmonary stromal cells

As depicted above, LPS signalling through TLR4 in pulmonary ECs involves at least the signal-transducing molecules MyD88, IRAK, and TRAF6 and activation of the transcription factor NF-κB [23]. Noulin et al. [9] showed that inhaled endotoxin-induced acute bronchoconstriction, TNF, IL-12p40, and KC production, protein leak, and neutrophil recruitment in the lung are abrogated in mice deficient for the adaptor molecule MyD88. MyD88 is involved in TLR, but also in IRAK-1-mediated IL-1R and -18R signalling. A role for IL-1 and IL-18 pathways in this response was excluded, as bronchoconstriction, inflammation, and protein leak were normal in IL-1R1 and caspase-1 (ICE)-deficient mice. Furthermore, using bone marrow chimera, it was shown that non-bone-marrow (BM)-derived radioresistant resident cells, probably ECs, were involved in sensing LPS to mediate the bronchoconstriction response, whereas the secretion of TNF and IL-12p40 in alveolar space was dependent on bone marrow-derived cells.

**Figure 2:**
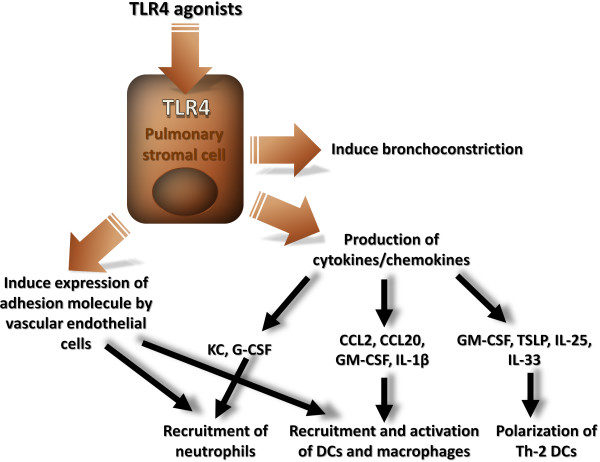
**Consequences of TLR4 activation on pulmonary SCs**. TLR4 signalling on SCs is required for early chemokine production and neutrophil and DC recruitment to the lungs, and direct bonchoconstriction, whereas robust cytokine production (IL-1, IL-6, IL-12p40, TNFα, etc.) is dependent of BM-derived cells. Moreover, the TLR4 signalling on pulmonary ECs induces a Th2 polarizing response, via the induction of Th2-inducing DCs.

To determine the role of respiratory ECs in the inflammatory response to inhaled endotoxin, Skerrett et al. [41] selectively inhibited NF-κB activation in the respiratory epithelium using a mutant IκB-α construct that functioned as a dominant negative inhibitor of NF-κB translocation (dnIκB-α). They developed two lines of transgenic mice in which expression of dnIκB-α was targeted to the distal airway epithelium using the human surfactant apoprotein C promoter. Transgene expression was localized to the epithelium of the terminal bronchioles and alveoli. After inhalation of LPS, nuclear translocation of NF-κB was evident in bronchiolar epithelium of nontransgenic but not of transgenic mice. This defect was associated with impaired neutrophilic lung inflammation 4 h after LPS challenge and diminished levels of TNF-α, IL-1β, macrophage inflammatory protein-2, and KC in lung homogenates. Expression of TNF-α within bronchiolar ECs and of VCAM-1 within peribronchiolar endothelial cells was reduced in transgenic animals. Thus targeted inhibition of NF-κB activation in distal airway ECs impaired the inflammatory response to inhaled LPS. Furthermore, Poynter et al. [42] reported that targeted expression of a dominant negative IκB-α in proximal airway ECs under the control of the rat CC10 promoter exhibited impaired airway inflammation in association with reduced levels of MIP-2 and TNF-α in BAL fluid after nasal challenge with LPS. The results of Skerrett et al. [41] and those of Poynter et al. [42] suggest that NF-κB activation in respiratory ECs contributes to the lung inflammatory response to inhaled LPS through the induction of proinflammatory cytokines, which in turn act to upregulate the expression of adhesion molecules on the vascular endothelium. Accordingly, we recently showed, using TLR4 chimeric mice [43], that the expression of TLR4 on SCs was crucial to recruit neutrophils and monocytes in response to LPS. This effect was likely to be mediated by several chemokines and by growth factors for neutrophils (KC, granulocyte colony-stimulating factor (G-CSF)), monocytes and DCs (C-C chemokine ligand-2 (CCL2), and CCL20). More than 70% of DCs recruited to the airways in response to LPS were inflammatory DCs as they expressed high levels of CD11b. These inflammatory DCs have been shown to derive from Ly6Chi blood monocytes, and to be recruited by the chemokine CCL2 under inflammatory conditions. Interestingly, we have observed an upregulation of CCL2 in the airways following LPS and house dust mite (HDM) administration [44,45]. The LPS- and the HDM-induced recruitment of inflammatory cells to the airways was abolished when SCs did not express TLR4 [43] (Figure 2). This result obtained with HDM was somewhat unexpected as, until very recently, it was unknown whether relevant environmental allergens such as HDM would be able to trigger TLRs. Phipps et al convincingly reported that the effects induced by HDM were reduced in MyD88^-/- ^and TLR4^-/- ^mice [46]. Using dynamic imaging of freshly explanted tracheal samples, we observed that LPS and HDM inhalation induced a rapid scanning behavior of tracheal MHCII^high ^DCs that depended on TLR4 expression by SCs. Such a scanning behaviour is typical of activated DCs and helps them to probe the mucosa for incoming antigens. Moreover, in response to LPS or HDM, TLR4^+ ^SCs produced DC-activating cytokines such as GM-CSF in the airways. This cytokine is likely to be involved in airway DC maturation, leading to their subsequent migration to the mediastinal lymph nodes, a process necessary for the activation of naïve T cells and the initiation of immune responses. This necessity of TLR4 expression in the initiation of Th2 responses in the airways was recently confirmed by Tan et al. in a similar chimeric mouse model [47]. It is important to note that the expression of TLRs by stromal cells is crucial in the control of immune response to a wide variety of antigens. Indeed, using MyD88 chimeric mice, Hajar et al. observed an important role of MyD88 in the early recruitment of inflammatory cells and in the control of bacterial infection [48].

## TLR4 signalling in pulmonary SCs polarizes the pulmonary immune response

In addition to their involvement in innate immune responses, the airway epithelium is also capable of driving the exacerbation of established allergic airway diseases by the production of pro-Th2 cytokines and chemokines such as IL-4, IL-13, TSLP, and TARC/CCL17 [49,50]. DCs, which densely line the airways, are critically involved in the pathogenesis of allergic diseases and are known to be potent inducers of CD4 T cell differentiation, expansion, and polarization [51,52]. However, the mechanism by which immature pulmonary DCs undergo maturation and become effector T cell-inducing antigen presenting cells (APCs) is unclear.

Using bone marrow chimeric mice to restrict TLR4 signalling to either the SC compartment (SC^+^HPC^-^) or the hematopoietic cell (HPC) compartment (SC^-^HPC^+^), we showed that TLR4 expression on lung radioresistant SCs, but not on DCs, is necessary and sufficient for DC activation in the lung and for priming of Th2 responses to HDM [43]. TLR4 triggering on SCs induced the activation of airway WT DCs as read out by CD86 and CD40 expression [53]. Moreover, in a WT animal exposed to LPS, DCs that had migrated to the draining lymph nodes were able to induce effector T cell responses characterized by the production of IL-17A and IFN-g. It was however intriguing to see that in chimeric mice lacking TLR4 expression on stromal cells, WT DCs in the airways were no longer able to induce affector T cell differentiation. The same held true when HDM was used instead of LPS. It is therefore very likely that TLR4-expressing stromal cells release factors that instruct airway DCs to induce a particular type of immune response. Such factors might include cytokines such as GM-CSF, known to induce DC activation [54], or other cytokines such as TSLP or IL-33 which might contribute to set the stage for Th2 response development [55,56]. In agreement with this, the absence of TLR4 on structural cells, but not on hematopoietic cells, prevented the development of HDM-driven allergic airway inflammation and the production of Th2 cytokines by mediastinal lymph node T cells. Interestingly, in the same mice, the levels of instructing cytokines were severely impaired. Interestingly, inhalation of a TLR4 antagonist to target ECs suppressed the salient features of asthma, including bronchial hyperreactivity. In a similar way, Th2 sensitization to inhaled ovalbumin (OVA), an antigen often used to induce asthma features in mice but often criticized for its content in LPS, seems to depend on recognition by stromal TLR4. When it comes to LPS, it is generally approved that the concentration of LPS determined the type of immune response induced, with high concentrations (LPS^high^) inducing Th1 responses and low concentrations (LPS^low^) inducing Th2 responses [57,58]. A recent study reported that using contaminated OVA contaminated with high levels of LPS, the stromal recognition of LPS by TLR4 led to a robust Th2 response, indicating that in the presence of higher concentrations of LPS, stromal cell expression of TLR4 is sufficient for Th2 sensitization [47]. In view of these results, one can wonder about the level of contamination of allergen preparation such as HDM extracts. When addressing this issue in our experiments showing a crucial role for stromal TLR4 expression in Th2 responses to HDM [43], we found that the degree of endotoxin contamination of HDM extract was in the subnanogram range, far below the dose previously reported to promote TH2 responses to OVA [57]. If HDM extracts contain such a low level of LPS contamination, why are they triggering TLR4? A very elegant study by Trompette et al. showed that Der p 2, one major allergen of the house dust mite Dermatophagoides pteronyssinus, was found to enhance the response of mouse bronchial ECs to endotoxin by acting as an MD2-like chaperone that promotes TLR4 signalling [59], providing an explanation to the profound proallergic innate response to HDM.

Altogether, these studies demonstrate that stromal cell TLR4 signalling is critically involved in Th2 but not Th1 sensitization to inhaled allergen [47]. Stromal TLR4 signalling leads to the maturation of Th2-inducing DCs that fail to produce proinflammatory cytokines or to upregulate the Th1-inducing Notch ligand Delta-4. Following intranasal administration of LPS or HDM into the airways, stromal cells upregulate mRNA expression or synthesis of TSLP, suggesting a stromal cell-dependent instruction of DCs in the priming of allergic Th2 responses (Figure 2).

## TLR4 signalling in non-infectious lung injury

The TLRs have well-established roles as pattern recognition receptors in acute infection [1,11]. More recent work has focused on the observation that the inflammatory response after trauma, hemorrhage, and ischemia-reperfusion injury has many similar features as that after acute infection [60,61]. Recently, Baudoin [61] reviewed these findings. For example, mice with TLR-4 mutations are resistant to both lipopolysaccharide and have an increased survival after experimental hemorrhagic shock [62]. Better survival has also been reported in experimental orthopaedic trauma and ischemia-reperfusion injury to the heart and lungs [60]. Experiments using TLR4 chimeric mice indicate that expression of functioning TLR on both marrow derived, immune cells and parenchymal tissue is necessary for noninfectious injury to occur [63]. Despite early suggestions that endotoxin mediate noninfectious tissue injury, it is now clear that TLR-4 can be activated by several ligands that are not derived from microbes [64] (Figure 2). These include high mobility group box 1 (HMGB1), a DNA-binding protein with proinflammatory properties, heparan sulfate, low-molecular-weight hyaluronan, fibrinogen, and heat shock proteins (HSPs). All these endogenous molecules are produced by or released from cells that are either severely stressed or dying and are called damage associated molecular pattern molecules (DAMPs). The release and sensing of these molecules would provide a mechanism for innate immune activation that is both independent and complementary to that produced by microbes alone. This is likely to amplify the immune response to infection in any body area where significant tissue injury occurs.

However, in some situations, the innate immune response, which evolved to limit the spread of infection, could become damaging. Ventilator-associated lung injury may be an example of such a situation [65]. Animals ventilated with elevated tidal volumes develop an acute lung injury that is characterized by the appearance within the lungs of acute inflammatory cells and the local production of proinflammatory mediators [66]. This may be caused by ventilator-induced activation of the innate immune system by the TLR-4 receptor. In a series of experiments with wild and TLR4-deficient mice, Hu et al showed that the acute lung injury, induced by ventilation, is reduced in animals that lack the Toll-like 4 receptor [65]. In the deficient animals, neutrophil accumulation was reduced as was the lung expression of TLR protein. In addition, in isolated lung preparations, they demonstrated that TLR-4 expression on both acute inflammatory cells and lung parenchymal cells was necessary for lung injury to develop. The results support and extend another recent publication on the effect of mechanical ventilation in TLR-4-deficient mice. In that study, TLR-4 knockouts were protected against the proinflammatory actions of mechanical ventilation [67]. However TLRs may also protect against acute lung injury in other situations. TLR2^-/-^TLR4^-/- ^dual knockout mice were more sensitive to both bleomycin and hyperoxia-induced acute lung injury and had increased mortality compared with wild-type controls [68].

## Conclusion

TLR4 signalling on SCs contributes to the lung inflammatory response to inhaled LPS through the induction of proinflammatory cytokines/chemokines, which in turn act to upregulate the expression of adhesion molecules on the vascular endothelium. TLR4 signalling on SCs is required for early chemokine production and neutrophil recruitment to the lungs, and direct bonchoconstriction. Moreover, the TLR4 signalling on pulmonary ECs induces a Th2 response by instructing airway DCs. The data reported in this review support the idea that a therapeutic strategy blocking TLR4 receptors might be effective in some forms of infectious and non infectious human lung diseases.

## List of abbreviations

APC: antigen presenting cells; BM: bone marrow; DAMP: damage associated molecular pattern molecule; DC: dendritic cells; dnIκB-α: dominant negative inhibitor of NF-κB translocation; EC: epithelial cell; ERK1/2: extracellular signal-regulated kinase 1/2; GM-CSF: granulocyte macrophage-colony-stimulating factor; HDM: house dust mite; HMGB1: high mobility group box 1; HPC: hematopoietic cell; HSP: heat shock protein; ICE mice: IL-1R1 and caspase-1-deficient mice; IL-1: interleukin-1; IRAK: IL-1R-associated kinase; LPS: lipopolysaccharide; MAPKs: mitogen-activated protein kinases; MIP-3α: macrophage inflammatory protein 3α; MyD88: myeloid differentiation factor 88; OVA: ovalbumin; SC: structural cell; TIRAP: Toll/IL-1R domain-containing adaptor protein; TLR: Toll-like receptor; TRAF6: tumor necrosis factor receptor-associated factor 6; TRAM: TRIF-related adaptor molecule; TRIF3: TIR domain-containing adaptor inducing IFN-β.

## Competing interests

The authors declare that they have no competing interests.

## Authors' contributions

FP, BL and HH contributed to drafting and revising the manuscript. All authors read and approved the final manuscript.

## Authors' information

FP has published in the fields of asthma, pulmonary hypertension and pulmonary inflammation. His research is mainly focused on the role of immunological pathomechanisms in the pulmonary vascular remodeling. BL and HH are the authors of over 100 papers dealing with the use of mouse models to study the pathogenesis of asthma and cancer related immunosuppression. The interest of their research group is on the role of antigen presenting dendritic cells in the initiation of the pulmonary immune response that ultimately leads to sensitization to antigens, applied to allergic disease, respiratory viruses and cancer immunotherapy.

## References

[B1] Medzhitov R Janeway C Jr Innate immunity N Engl J Med 2000 343 5 338 344 10.1056/NEJM200008033430506 10922424

[B2] Ghosh S May MJ Kopp EB NF-kappa B and Rel proteins: evolutionarily conserved mediators of immune responses Annu Rev Immunol 1998 16 225 260 10.1146/annurev.immunol.16.1.225 9597130

[B3] Medzhitov R Preston-Hurlburt P Janeway CA Jr A human homologue of the Drosophila Toll protein signals activation of adaptive immunity Nature 1997 388 6640 394 397 10.1038/41131 9237759

[B4] Beutler B TLR4 as the mammalian endotoxin sensor Curr Top Microbiol Immunol 2002 270 109 120 10.1007/978-3-642-59430-4_7 12467247

[B5] Takeuchi O Akira S MyD88 as a bottle neck in Toll/IL-1 signaling Curr Top Microbiol Immunol 2002 270 155 167 10.1007/978-3-642-59430-4_10 12467250

[B6] O'Neill LA Signal transduction pathways activated by the IL-1 receptor/toll-like receptor superfamily Curr Top Microbiol Immunol 2002 270 47 61 10.1007/978-3-642-59430-4_3 12467243

[B7] Hoffmann E Dittrich-Breiholz O Holtmann H Kracht M Multiple control of interleukin-8 gene expression J Leukoc Biol 2002 72 5 847 855 12429706

[B8] Gribar SC Richardson WM Sodhi CP Hackam DJ No longer an innocent bystander: epithelial toll-like receptor signaling in the development of mucosal inflammation Mol Med 2008 14 9-10 645 659 18584047 10.2119/2008-00035.Gribar PMC2435494

[B9] Noulin N Quesniaux VF Schnyder-Candrian S Schnyder B Maillet I Robert T Vargaftig BB Ryffel B Couillin I Both hemopoietic and resident cells are required for MyD88-dependent pulmonary inflammatory response to inhaled endotoxin J Immunol 2005 175 10 6861 6869 16272344 10.4049/jimmunol.175.10.6861

[B10] Kawai T Adachi O Ogawa T Takeda K Akira S Unresponsiveness of MyD88-deficient mice to endotoxin Immunity 1999 11 1 115 122 10.1016/S1074-7613(00)80086-2 10435584

[B11] Akira S Mammalian Toll-like receptors Curr Opin Immunol 2003 15 1 5 11 10.1016/S0952-7915(02)00013-4 12495726

[B12] Yamamoto M Sato S Mori K Hoshino K Takeuchi O Takeda K Akira S Cutting edge: a novel Toll/IL-1 receptor domain-containing adapter that preferentially activates the IFN-beta promoter in the Toll-like receptor signaling J Immunol 2002 169 12 6668 6672 12471095 10.4049/jimmunol.169.12.6668

[B13] Hoebe K Du X Georgel P Janssen E Tabeta K Kim SO Goode J Lin P Mann N Mudd S Identification of Lps2 as a key transducer of MyD88-independent TIR signalling Nature 2003 424 6950 743 748 10.1038/nature01889 12872135

[B14] Fitzgerald KA Rowe DC Barnes BJ Caffrey DR Visintin A Latz E Monks B Pitha PM Golenbock DT LPS-TLR4 signaling to IRF-3/7 and NF-kappaB involves the toll adapters TRAM and TRIF J Exp Med 2003 198 7 1043 1055 10.1084/jem.20031023 14517278 PMC2194210

[B15] Yamamoto M Sato S Hemmi H Uematsu S Hoshino K Kaisho T Takeuchi O Takeda K Akira S TRAM is specifically involved in the Toll-like receptor 4-mediated MyD88-independent signaling pathway Nat Immunol 2003 4 11 1144 1150 10.1038/ni986 14556004

[B16] Fitzgerald KA Palsson-McDermott EM Bowie AG Jefferies CA Mansell AS Brady G Brint E Dunne A Gray P Harte MT Mal (MyD88-adapter-like) is required for Toll-like receptor-4 signal transduction Nature 2001 413 6851 78 83 10.1038/35092578 11544529

[B17] Yamamoto M Sato S Hemmi H Sanjo H Uematsu S Kaisho T Hoshino K Takeuchi O Kobayashi M Fujita T Essential role for TIRAP in activation of the signalling cascade shared by TLR2 and TLR4 Nature 2002 420 6913 324 329 10.1038/nature01182 12447441

[B18] Horng T Barton GM Medzhitov R TIRAP: an adapter molecule in the Toll signaling pathway Nat Immunol 2001 2 9 835 841 10.1038/ni0901-835 11526399

[B19] Horng T Barton GM Flavell RA Medzhitov R The adaptor molecule TIRAP provides signalling specificity for Toll-like receptors Nature 2002 420 6913 329 333 10.1038/nature01180 12447442

[B20] Yamamoto M Sato S Hemmi H Hoshino K Kaisho T Sanjo H Takeuchi O Sugiyama M Okabe M Takeda K Role of adaptor TRIF in the MyD88-independent toll-like receptor signaling pathway Science 2003 301 5633 640 643 10.1126/science.1087262 12855817

[B21] Hirotani T Yamamoto M Kumagai Y Uematsu S Kawase I Takeuchi O Akira S Regulation of lipopolysaccharide-inducible genes by MyD88 and Toll/IL-1 domain containing adaptor inducing IFN-beta Biochem Biophys Res Commun 2005 328 2 383 392 10.1016/j.bbrc.2004.12.184 15694359

[B22] Yamamoto M Yaginuma K Tsutsui H Sagara J Guan X Seki E Yasuda K Yamamoto M Akira S Nakanishi K ASC is essential for LPS-induced activation of procaspase-1 independently of TLR-associated signal adaptor molecules Genes Cells 2004 9 11 1055 1067 10.1111/j.1365-2443.2004.00789.x 15507117

[B23] Guillot L Medjane S Le-Barillec K Balloy V Danel C Chignard M Si-Tahar M Response of human pulmonary epithelial cells to lipopolysaccharide involves Toll-like receptor 4 (TLR4)-dependent signaling pathways: evidence for an intracellular compartmentalization of TLR4 J Biol Chem 2004 279 4 2712 2718 14600154 10.1074/jbc.M305790200

[B24] Hume DA Underhill DM Sweet MJ Ozinsky AO Liew FY Aderem A Macrophages exposed continuously to lipopolysaccharide and other agonists that act via toll-like receptors exhibit a sustained and additive activation state BMC Immunol 2001 2 11 10.1186/1471-2172-2-11 11686851 PMC58839

[B25] Guha M Mackman N LPS induction of gene expression in human monocytes Cell Signal 2001 13 2 85 94 10.1016/S0898-6568(00)00149-2 11257452

[B26] Zarember KA Godowski PJ Tissue expression of human Toll-like receptors and differential regulation of Toll-like receptor mRNAs in leukocytes in response to microbes, their products, and cytokines J Immunol 2002 168 2 554 561 11777946 10.4049/jimmunol.168.2.554

[B27] Cario E Brown D McKee M Lynch-Devaney K Gerken G Podolsky DK Commensal-associated molecular patterns induce selective toll-like receptor-trafficking from apical membrane to cytoplasmic compartments in polarized intestinal epithelium Am J Pathol 2002 160 1 165 173 10.1016/S0002-9440(10)64360-X 11786410 PMC1867149

[B28] Hornef MW Frisan T Vandewalle A Normark S Richter-Dahlfors A Toll-like receptor 4 resides in the Golgi apparatus and colocalizes with internalized lipopolysaccharide in intestinal epithelial cells J Exp Med 2002 195 5 559 570 10.1084/jem.20011788 11877479 PMC2193765

[B29] Wolfs TG Buurman WA van Schadewijk A de Vries B Daemen MA Hiemstra PS van 't Veer C In vivo expression of Toll-like receptor 2 and 4 by renal epithelial cells: IFN-gamma and TNF-alpha mediated up-regulation during inflammation J Immunol 2002 168 3 1286 1293 11801667 10.4049/jimmunol.168.3.1286

[B30] Uehara A Sugawara S Tamai R Takada H Contrasting responses of human gingival and colonic epithelial cells to lipopolysaccharides, lipoteichoic acids and peptidoglycans in the presence of soluble CD14 Med Microbiol Immunol 2001 189 4 185 192 10.1007/s004300100063 11599788

[B31] Sha Q Truong-Tran AQ Plitt JR Beck LA Schleimer RP Activation of airway epithelial cells by toll-like receptor agonists Am J Respir Cell Mol Biol 2004 31 3 358 364 10.1165/rcmb.2003-0388OC 15191912

[B32] Andonegui G Bonder CS Green F Mullaly SC Zbytnuik L Raharjo E Kubes P Endothelium-derived Toll-like receptor-4 is the key molecule in LPS-induced neutrophil sequestration into lungs J Clin Invest 2003 111 7 1011 1020 12671050 10.1172/JCI16510 PMC152584

[B33] Schulz C Farkas L Wolf K Kratzel K Eissner G Pfeifer M Differences in LPS-induced activation of bronchial epithelial cells (BEAS-2B) and type II-like pneumocytes (A-549) Scand J Immunol 2002 56 3 294 302 10.1046/j.1365-3083.2002.01137.x 12193231

[B34] Simpson JC Niven RM Pickering CA Oldham LA Fletcher AM Francis HC Comparative personal exposures to organic dusts and endotoxin Ann Occup Hyg 1999 43 2 107 115 10206039

[B35] Thieblemont N Thieringer R Wright SD Innate immune recognition of bacterial lipopolysaccharide: dependence on interactions with membrane lipids and endocytic movement Immunity 1998 8 6 771 777 10.1016/S1074-7613(00)80582-8 9655491

[B36] Vasselon T Hailman E Thieringer R Detmers PA Internalization of monomeric lipopolysaccharide occurs after transfer out of cell surface CD14 J Exp Med 1999 190 4 509 521 10.1084/jem.190.4.509 10449522 PMC2195596

[B37] Hedlund M Frendeus B Wachtler C Hang L Fischer H Svanborg C Type 1 fimbriae deliver an LPS- and TLR4-dependent activation signal to CD14-negative cells Mol Microbiol 2001 39 3 542 552 10.1046/j.1365-2958.2001.02205.x 11169097

[B38] Pugin J Schurer-Maly CC Leturcq D Moriarty A Ulevitch RJ Tobias PS Lipopolysaccharide activation of human endothelial and epithelial cells is mediated by lipopolysaccharide-binding protein and soluble CD14 Proc Natl Acad Sci USA 1993 90 7 2744 2748 10.1073/pnas.90.7.2744 7681988 PMC46172

[B39] Diamond G Legarda D Ryan LK The innate immune response of the respiratory epithelium Immunol Rev 2000 173 27 38 10.1034/j.1600-065X.2000.917304.x 10719665

[B40] Becker MN Diamond G Verghese MW Randell SH CD14-dependent lipopolysaccharide-induced beta-defensin-2 expression in human tracheobronchial epithelium J Biol Chem 2000 275 38 29731 29736 10.1074/jbc.M000184200 10882713

[B41] Skerrett SJ Liggitt HD Hajjar AM Ernst RK Miller SI Wilson CB Respiratory epithelial cells regulate lung inflammation in response to inhaled endotoxin Am J Physiol Lung Cell Mol Physiol 2004 287 1 L143 152 10.1152/ajplung.00030.2004 15047567

[B42] Poynter ME Irvin CG Janssen-Heininger YM A prominent role for airway epithelial NF-kappa B activation in lipopolysaccharide-induced airway inflammation J Immunol 2003 170 12 6257 6265 12794158 10.4049/jimmunol.170.12.6257

[B43] Hammad H Chieppa M Perros F Willart MA Germain RN Lambrecht BN House dust mite allergen induces asthma via Toll-like receptor 4 triggering of airway structural cells Nat Med 2009 15 4 410 416 10.1038/nm.1946 19330007 PMC2789255

[B44] Robays LJ Maes T Lebecque S Lira SA Kuziel WA Brusselle GG Joos GF Vermaelen KV Chemokine receptor CCR2 but not CCR5 or CCR6 mediates the increase in pulmonary dendritic cells during allergic airway inflammation J Immunol 2007 178 8 5305 5311 17404315 10.4049/jimmunol.178.8.5305

[B45] Geissmann F Jung S Littman DR Blood monocytes consist of two principal subsets with distinct migratory properties Immunity 2003 19 1 71 82 10.1016/S1074-7613(03)00174-2 12871640

[B46] Phipps S Lam CE Kaiko GE Foo SY Collison A Mattes J Barry J Davidson S Oreo K Smith L Toll/IL-1 signaling is critical for house dust mite-specific helper T cell type 2 and type 17 [corrected] responses Am J Respir Crit Care Med 2009 179 10 883 893 10.1164/rccm.200806-974OC 19246719

[B47] Tan AM Chen HC Pochard P Eisenbarth SC Herrick CA Bottomly HK TLR4 signaling in stromal cells is critical for the initiation of allergic Th2 responses to inhaled antigen J Immunol 2010 184 7 3535 3544 10.4049/jimmunol.0900340 20194715

[B48] Hajjar AM Harowicz H Liggitt HD Fink PJ Wilson CB Skerrett SJ An essential role for non-bone marrow-derived cells in control of Pseudomonas aeruginosa pneumonia Am J Respir Cell Mol Biol 2005 33 5 470 475 10.1165/rcmb.2005-0199OC 16100080 PMC2715354

[B49] Luster AD Tager AM T-cell trafficking in asthma: lipid mediators grease the way Nat Rev Immunol 2004 4 9 711 724 10.1038/nri1438 15343370

[B50] Ying S O'Connor B Ratoff J Meng Q Mallett K Cousins D Robinson D Zhang G Zhao J Lee TH Thymic stromal lymphopoietin expression is increased in asthmatic airways and correlates with expression of Th2-attracting chemokines and disease severity J Immunol 2005 174 12 8183 8190 15944327 10.4049/jimmunol.174.12.8183

[B51] Lambrecht BN Dendritic cells and the regulation of the allergic immune response Allergy 2005 60 3 271 282 10.1111/j.1398-9995.2005.00708.x 15679711

[B52] Vermaelen KY Carro-Muino I Lambrecht BN Pauwels RA Specific migratory dendritic cells rapidly transport antigen from the airways to the thoracic lymph nodes J Exp Med 2001 193 1 51 60 10.1084/jem.193.12.F51 11136820 PMC2195883

[B53] Vermaelen K Pauwels R Pulmonary dendritic cells Am J Respir Crit Care Med 2005 172 5 530 551 10.1164/rccm.200410-1384SO 15879415

[B54] Ritz SA Cundall MJ Gajewska BU Swirski FK Wiley RE Alvarez D Coyle AJ Stampfli MR Jordana M The lung cytokine microenvironment influences molecular events in the lymph nodes during Th1 and Th2 respiratory mucosal sensitization to antigen in vivo Clin Exp Immunol 2004 138 2 213 220 10.1111/j.1365-2249.2004.02618.x 15498029 PMC1809215

[B55] Liu YJ Soumelis V Watanabe N Ito T Wang YH Malefyt Rde W Omori M Zhou B Ziegler SF TSLP: an epithelial cell cytokine that regulates T cell differentiation by conditioning dendritic cell maturation Annu Rev Immunol 2007 25 193 219 10.1146/annurev.immunol.25.022106.141718 17129180

[B56] Ziegler SF Artis D Sensing the outside world: TSLP regulates barrier immunity Nat Immunol 2010 11 4 289 293 10.1038/ni.1852 20300138 PMC2924817

[B57] Eisenbarth SC Piggott DA Huleatt JW Visintin I Herrick CA Bottomly K Lipopolysaccharide-enhanced, toll-like receptor 4-dependent T helper cell type 2 responses to inhaled antigen J Exp Med 2002 196 12 1645 1651 10.1084/jem.20021340 12486107 PMC2196061

[B58] Kim YK Oh SY Jeon SG Park HW Lee SY Chun EY Bang B Lee HS Oh MH Kim YS Airway exposure levels of lipopolysaccharide determine type 1 versus type 2 experimental asthma J Immunol 2007 178 8 5375 5382 17404323 10.4049/jimmunol.178.8.5375

[B59] Trompette A Divanovic S Visintin A Blanchard C Hegde RS Madan R Thorne PS Wills-Karp M Gioannini TL Weiss JP Allergenicity resulting from functional mimicry of a Toll-like receptor complex protein Nature 2009 457 7229 585 588 10.1038/nature07548 19060881 PMC2843411

[B60] Kaczorowski DJ Mollen KP Edmonds R Billiar TR Early events in the recognition of danger signals after tissue injury J Leukoc Biol 2008 83 3 546 552 18032691 10.1189/jlb.0607374

[B61] Baudouin S Innate immune defense on the attack in acute lung injury Crit Care Med 2010 38 1 328 329 10.1097/CCM.0b013e3181b3a81e 20023487

[B62] Lv T Shen X Shi Y Song Y TLR4 is essential in acute lung injury induced by unresuscitated hemorrhagic shock J Trauma 2009 66 1 124 131 10.1097/TA.0b013e318181e555 19131815

[B63] Mollen KP Levy RM Prince JM Hoffman RA Scott MJ Kaczorowski DJ Vallabhaneni R Vodovotz Y Billiar TR Systemic inflammation and end organ damage following trauma involves functional TLR4 signaling in both bone marrow-derived cells and parenchymal cells J Leukoc Biol 2008 83 1 80 88 17925504 10.1189/jlb.0407201

[B64] Beg AA Endogenous ligands of Toll-like receptors: implications for regulating inflammatory and immune responses Trends Immunol 2002 23 11 509 512 10.1016/S1471-4906(02)02317-7 12401394

[B65] Hu G Malik AB Minshall RD Toll-like receptor 4 mediates neutrophil sequestration and lung injury induced by endotoxin and hyperinflation Crit Care Med 2010 38 1 194 201 10.1097/CCM.0b013e3181bc7c17 19789446 PMC3739989

[B66] Tremblay LN Slutsky AS Ventilator-induced lung injury: from the bench to the bedside Intensive Care Med 2006 32 1 24 33 10.1007/s00134-005-2817-8 16231069

[B67] Vaneker M Joosten LA Heunks LM Snijdelaar DG Halbertsma FJ van Egmond J Netea MG van der Hoeven JG Scheffer GJ Low-tidal-volume mechanical ventilation induces a toll-like receptor 4-dependent inflammatory response in healthy mice Anesthesiology 2008 109 3 465 472 10.1097/ALN.0b013e318182aef1 18719444

[B68] Jiang D Liang J Fan J Yu S Chen S Luo Y Prestwich GD Mascarenhas MM Garg HG Quinn DA Regulation of lung injury and repair by Toll-like receptors and hyaluronan Nat Med 2005 11 11 1173 1179 10.1038/nm1315 16244651

